# Optimizing the composition of a synthetic cellulosome complex for the hydrolysis of softwood pulp: identification of the enzymatic core functions and biochemical complex characterization

**DOI:** 10.1186/s13068-018-1220-y

**Published:** 2018-08-09

**Authors:** Benedikt Leis, Claudia Held, Björn Andreeßen, Wolfgang Liebl, Sigrid Graubner, Louis-Philipp Schulte, Wolfgang H. Schwarz, Vladimir V. Zverlov

**Affiliations:** 1 Department of Microbiology, Technische Universität München, TUM School of Life Sciences Weihenstephan, Emil-Ramann-Str. 4, 85354 Freising, Germany; 2 Institute of Molecular Genetics, Russian Academy of Science, Kurchatov Sq. 2, Moscow, 123182 Russia; 3 Present Address: Fraunhofer Institute for Molecular Biology and Applied Ecology IME, Winchester Str. 2, 35394 Gießen, Germany

**Keywords:** *Clostridium thermocellum*, Cellulosome, Screening, Synthetic cellulase complex, Softwood, Cellulose

## Abstract

**Background:**

The development of efficient cellulase blends is a key factor for cost-effectively valorizing biomass in a new bio-economy. Today, the enzymatic hydrolysis of plant-derived polysaccharides is mainly accomplished with fungal cellulases, whereas potentially equally effective cellulose-degrading systems from bacteria have not been developed. Particularly, a thermostable multi-enzyme cellulase complex, the cellulosome from the anaerobic cellulolytic bacterium *Clostridium thermocellum* is promising of being applied as cellulolytic nano-machinery for the production of fermentable sugars from cellulosic biomass.

**Results:**

In this study, 60 cellulosomal components were recombinantly produced in *E. coli* and systematically permuted in synthetic complexes to study the function–activity relationship of all available enzymes on Kraft pulp from pine wood as the substrate. Starting from a basic exo/endoglucanase complex, we were able to identify additional functional classes such as mannanase and xylanase for optimal activity on the substrate. Based on these results, we predicted a synthetic cellulosome complex consisting of seven single components (including the scaffoldin protein and a β-glucosidase) and characterized it biochemically. We obtained a highly thermostable complex with optimal activity around 60–65 °C and an optimal pH in agreement with the optimum of the native cellulosome (pH 5.8). Remarkably, a fully synthetic complex containing 47 single cellulosomal components showed comparable activity with a commercially available fungal enzyme cocktail on the softwood pulp substrate.

**Conclusions:**

Our results show that synthetic bacterial multi-enzyme complexes based on the cellulosome of *C. thermocellum* can be applied as a versatile platform for the quick adaptation and efficient degradation of a substrate of interest.

**Electronic supplementary material:**

The online version of this article (10.1186/s13068-018-1220-y) contains supplementary material, which is available to authorized users.

## Background

Cellulose and hemicellulose from plants are the most abundant carbohydrates on earth and a ubiquitous and regenerative resource for the generation of second-generation biofuels. Substrate depolymerization into fermentable sugars is one of the limiting steps within the value chain of the biorefinery process [1, 2]. Due to the recalcitrant nature of this substrate, effective and cost-competitive enzyme mixtures for the hydrolysis of cellulose are highly demanded.

The extracellular multi-enzyme complex of the anaerobic bacterium *Clostridium thermocellum* is an effective cellulase nano-machinery to hydrolyze crystalline cellulose from plant-derived biomass [3–5]. Its effectivity is due to the co-localization of many different enzymatic functions needed to act synergistically on the highly complex matrix of polysaccharides for most efficient breakdown to sugars [6].

For the development of a competitive synthetic cellulase complex, a higher effectiveness of the cellulosomes than existing enzyme cocktails is needed [7]. When the components are separately produced recombinantly, one of the major advantages over fungal enzyme cocktails is the possibility to quickly adapt the composition of synthetic cellulase complexes by selectively adding new enzymatic functions or to change the stoichiometry of components added. Another advantage of the bacterial components from thermophiles is their higher temperature optimum compared to the fungal enzymes, a key feature to increase solubility of substrate and by-products, to increase diffusion rates, and to decrease viscosity. A higher process stability due to reduced microbial contamination risks is a further benefit [8]. However, despite many decades of research in this field, the commercial use of these native enzyme complexes is mainly hampered by the low production yield from anaerobic bacteria [9].

The *C. thermocellum* cellulosome is characterized by the binding of over 70 catalytic and non-catalytic protein components on a scaffoldin protein CipA [10]. This binding is mediated through a very strong protein–protein interaction between the dockerins located on each cellulosomal component, and one of the nine cohesin modules of CipA. Native and recombinantly produced cellulosomal enzymes have been combined in vitro on a scaffoldin and form complexes stoichiometrically in statistical distribution [7]. There are numerous studies that show the influence of different enzymatic functions on the complex effectivity, such as the presence of auxiliary enzymes [11], enzyme additives [12], enzymatic processivity modes [13], enzymatic diversity and stoichiometry [14–16]. However, to the best of our knowledge, synthetic cellulosomal cellulases have so far been unsuccessful in reaching the activity of commercial cellulase blends.

In this study, we show the rapid adaptation of a fully synthetic cellulosome complex on an industrial substrate based on delignified softwood from Kraft pulp process and present a screening strategy to identify enzymatic functions necessary within the cellulosome complex to enhance substrate degradation. To employ this strategy, over 60 cellulosomal proteins from *C. thermocellum* containing a dockerin module were cloned and successfully expressed, including cellulases, hemicellulases, structural proteins and proteins with unknown function. Mixtures of these enzymes were bound to a recombinant scaffoldin and systematically tested for substrate degradation efficiency.

Our approach underscores the versatility and advantage of a simple and fast adaptation strategy using fully recombinant cellulosome complexes. This strategy reduces the complexity of random combinations and may help to develop cost-effective and efficient bacterial cellulase mixtures in the future.

## Methods

### Strains and media

*Clostridium thermocellum* (also referred to as *Ruminiclostridium thermocellum*) strains DSM1313 and mutant strain SM901 (strain devoid of *cip*A scaffoldin-encoding gene, also referred to as SM1 [17]) were grown at 60 °C in prereduced GS-2 [18] medium for liquid cultures containing 0.5% (w/v) cellobiose, Whatman filter paper (both purchased at Sigma-Aldrich, St. Louis, USA) or softwood pulp. Bleached and delignified Kraft pulp from pine (softwood) was a generous gift from Michael Duetsch from UPM-Kymmene Oy (Finland). Strains *Escherichia coli* DH10B and DH5α were used for cloning. *E. coli* strains for protein expression were BL21 Star (DE3) (Invitrogen, Carlsbad, USA), Arctic Express (DE3), BL21 Codon Plus (DE3) RIPL (Agilent Technologies, Santa Clara, USA) and Rosetta-gami B (DE3) (Novagen–Merck, Darmstadt, Germany). Cells were grown in lysogeny broth (LB) containing 100 µg/mL ampicillin for pET21a(+) plasmids and 50 µg/mL kanamycin for pET24(+) plasmids.

### DNA cloning

DNA fragments were assembled with Gibson Assembly Master Mix (NEB, Ipswich, USA). QIAprep Spin Miniprep kit and PCR purification kit (Qiagen, Hilden, Germany) were used for the purification of recombinant plasmids and PCR products. DNA sequences encoding recombinant protein constructs were PCR amplified with Phusion DNA polymerase (NEB) and cloned without the predicted N-terminal signal peptides as identified using the SignalP 4.0 server [19]. Oligonucleotides are listed in Additional file 1. The amplicons were digested and ligated in frame into the multiple cloning site of plasmids pET21a(+) or pET24(+). The genes encoding for Cel9-44J (Clo1313_1604), Cel124A (Clo1313_1786), Cel9K (Clo1313_1809), and Cel48S (Clo1313_2747) were optimized in *E. coli* codon usage by Eurofins (Ebersberg, Germany). The cellulosomal scaffoldin protein CipA8 was synthesized in optimized *E. coli* codon usage and DNA sequence, including eight cohesins, the carbohydrate-binding module CBM3 and the C-terminal X-module from *C. thermocellum* WP_020458017.1 lacking Coh6 and dockerin type II [13]. Correct cloning was verified by sequencing (MWG-Eurofins, Ebersberg, Germany).

### Protein purification

For protein expression, *E. coli* cells were grown at 37 °C, room temperature (RT) or lower temperatures in LB medium containing chloramphenicol (34 µg/mL for BL21 Codon Plus), gentamycin (20 µg/mL for Arctic Express) and kanamycin (25 µg/mL for Rosetta-gami B). Heterologous protein expression was induced by the addition of 1 mM isopropyl-β-d-thiogalactopyranoside (IPTG) to an exponentially growing culture. After further growth for 4 h (or overnight incubation with Arctic Express) the cells were harvested by centrifugation at 3440×*g* for 10 min at 4 °C. Heterologously expressed proteins and the native cellulosome from *C. thermocellum* were prepared as previously described [13]. Before cell lysis, Roche cOmplete Mini EDTA-free protease inhibitor cocktail tablets (purchased from Sigma-Aldrich) were added. The cells were resuspended in 20 mL lysis buffer (50 mM MOPS, pH 7.3, 100 mM NaCl, 10 mM CaCl_2_, 20 mM imidazole) with the addition of 10 mg/mL lysozyme (AppliChem, Darmstadt, Germany) and Roche cOmplete Mini EDTA-free protease inhibitor cocktail tablets (purchased from Sigma-Aldrich). After incubation for 30 min on ice, the cells were sonified twice with Sonifier UP 200S (Hielscher, Teltow, Germany) set at amplitude 60%, interval 0.25 for 4 min. After centrifugation (18,000 rpm, 20 min, 4 °C) the supernatant was loaded onto an immobilized metal HisTrap affinity column (IMAC) (GE Healthcare, Munich, Germany) and eluted with 0.5 M imidazole, 50 mM MOPS, pH 7.3, 100 mM NaCl, and 10 mM CaCl_2_. All enzyme preparations were heat treated for 15 min at 60 °C and precipitates were separated from the supernatant by centrifugation (13,000 rpm for 10 min at RT). The proteins were examined by sodium dodecyl sulfate-polyacrylamide gel electrophoresis (SDS-PAGE) and stained with Coomassie brilliant blue R-250. Protein concentrations were determined using Pierce BCA protein quantification kit (Thermo Fisher Scientific).

### Complex assembly

Synthetic cellulosome complexes were assembled in complex assembly buffer for 1 h at RT with a fixed concentration of the scaffoldin protein CipA8 comprising eight type I cohesins and an amount of single enzymes equimolar with the available cohesins. Following complex stoichiometries were used: 0.87 nmol of the CipA8 corresponds to free cohesin concentrations of 6.75 nmol in a standard complexation reaction of 0.55 mL. For the fully loaded SKL complex, 2.25 nmol of each component Cel48S, Cel9K and Cel5L was mixed (each representing 33.3% stoichiometric binding). A not fully loaded SKL complex is stochastically populated by 75% of the available cohesins with an equimolar mix of Cel48S, Cel9K and Cel5L (1.69 nmol of each). For the SKLM or SKLY complexes, 1.97 nmol of Cel48S, Cel9K and Cel5L (in sum binding 87.5% of all available cohesins) were mixed with 0.84 nmol of Man26A or Xyn10Y (equals 12.5% loading). Pentavalent SKLMY was assembled with 1.69 nmol of the cellulases (Cel48S, Cel9K, Cel5L, each binding 25%) and 0.84 nmol of each additional protein (Man26A, Xyn10Y, each binding 12.5%). Fully recombinant complexes were purified from non-complexed proteins by size-exclusion chromatography on a Superdex 200 10/300 GL column (GE Healthcare, Little Chalfont, UK) equilibrated with a buffer containing 50 mM MOPS, pH 7.3, 0.5 M NaCl, and 20 mM CaCl_2_. Size-exclusion chromatography was carried out in an ÄKTA Purifier (GE Healthcare, Munich, Germany). The column was developed with the same buffer at a flow rate of 0.5 mL/min. Fractions of 1 mL were collected and concentrated with Vivaspin 500 columns with a cutoff of 50 kDa. Protein concentrations were determined by the BCA method using BSA as the standard. The complexation reaction in 20 µL final volume was visualized on 6% native PAGE as described elsewhere [13]. The influence of the substrate on complex formation was studied as follows: as complexation master mix, 20 µg of CipA8 was bound on 1.25 mg substrate, followed by the addition of 200 µg native enzyme extract (from scaffoldin protein-devoid mutant SM901 [17]). Cellulosomes from unbound cellulosomal components on synthetic CipA8 were assembled as described by Krauss et al. [7].

### Substrate binding of CipA8

For binding analysis of a recombinant CipA8 on the insoluble substrate, 20 µg of the scaffoldin protein was mixed with 1.25 mg softwood pulp in 250 µL 0.1 M MOPS buffer (pH 6.5), containing 50 mM NaCl and 20 mM CaCl_2_. After 5 min of binding at RT, the reaction was spun down and the pellet washed three times with buffer. The reaction mixture was finally mixed with 20 µL of 4× concentrated denaturing protein loading dye and the supernatant was completely loaded on a SDS gel.

### Enzymatic assays

Enzymatic reactions using cellulosome complexes were performed under standard reaction conditions at 60 °C in a total volume of 0.5 mL. The reaction buffer contained 0.1 M MOPS, pH 6.5, 50 mM NaCl, and 10 mM CaCl_2_. Cellic CTec2 (Novozymes A/S, Bagsværd, Denmark; from Sigma-Aldrich, St. Louis, USA) was incubated at 50 °C in 0.1 M MES and 50 mM CaCl_2_ buffered at pH 5. The activity of synthetic cellulosome complexes was measured on 0.25–0.5% (w/v) microcrystalline cellulose (Avicel, from Sigma-Aldrich) and micronized softwood pulp (UPM-Kymmene, Finland). Softwood was treated with an Ultra Turrax homogenizer (Ika, Staufen, Germany) until the homogeneously micronized substrate could be pipetted using wide-bored tips. To avoid inhibition of the complexed cellulases by cellobiose, β-glucosidase BglT (TT_P0042) from *Thermus thermophilus* [20] or CglT (Q60026_THEBR) from *Thermoanaerobacter brockii* [21] was added to a final concentration of 6 µg/mL. The presence of d-glucose in reaction mixtures was determined with the d-glucose HK assay kit (Megazyme, Wicklow, Ireland). Reducing sugars released from the substrates were quantified using the 3,5-dinitrosalicylic acid method [22]. One enzymatic unit liberates 1 µmol of glucose equivalent per minute.

### Two-step acid substrate hydrolysis

Substrate analysis with a two-step sulfuric acid hydrolysis was carried out as follows: 100 mg of substrate was hydrolyzed by adding 7 mL of 2% sulfuric acid, incubated for 1 h at 30 °C and mixed by homogenization every 15 min. Then, the mixture was incubated at 121 °C for 1 h. After cooling and centrifugation, the supernatant was stored at 8 °C. The pellet was dried overnight and hydrolyzed by adding 600 µL of 72% sulfuric acid, incubated at 35 °C for 1 h, mixed with 8 mL water, and autoclaved at 121 °C for 1 h. The mixture was centrifuged; the second pellet (acid-insoluble lignin and inorganic constituents) was weighed. The supernatant (approx. 8.6 mL) was mixed with 7 mL from the first hydrolysis and filled to 50 mL final volume. For neutralization of the acidic reaction mixture, calcium carbonate was added until the pH was > 5. The amount of acid-hydrolyzed sugar monomers was determined by the glucose detection kit and DNSA assay. Each hydrolysis was carried out in duplicates.

## Results

**Fig. 1:**
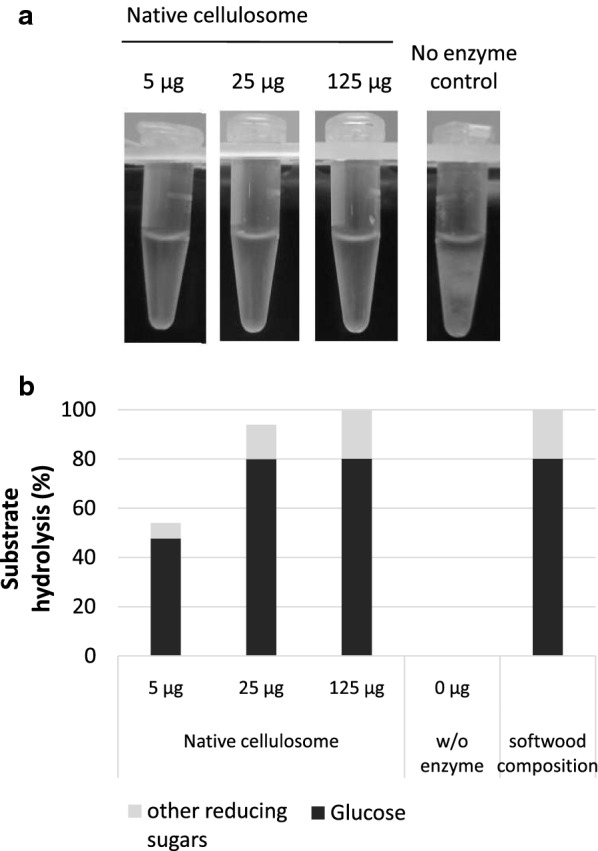
Preliminary assessment of enzymatic hydrolysis of softwood pulp. **a** Hydrolysis reaction of native cellulosome preparation on 0.25% (w/v) softwood after 24 h at 60 °C (5, 25 and 125 µg of enzyme per 1.25 mg softwood in 0.5 mL). **b** Measurement of released glucose (black bar) and reducing sugar ends (as determined with the DNSA assay, gray bars) from the substrate at different enzyme loadings (average values from triplicate measurements). The softwood composition was determined using a two-step protocol using sulfuric acid as hydrolysis agent

*Clostridium thermocellum* was able to grow on Kraft softwood fibers over several days under anaerobic conditions (data not shown). Cellulosomes from *C. thermocellum* cultures were purified and their ability to degrade softwood fibers was verified by visual inspection and quantification of hydrolytic products (Fig. 1a, b). According to the results from two-step acid hydrolysis, softwood polysaccharides from kraft pulp contain approx. 80% β-d-glucose and 20% other reducing sugars (Fig. 1b). Within 24 h, kraft softwood fibers were completely hydrolysed to soluble sugars by 25 µg native cellulosome complex including β-glucosidase per 1.25 mg substrate (substrate to enzyme ratio of 1:50).

In a previous study, a nonavalent synthetic cellulosome complex (nine different single cellulase components on recombinant CipA8 scaffoldin protein) showed half of the activity of the native cellulosome complex from *C. thermocellum* on microcrystalline cellulose Avicel [13]. The substrate softwood pulp contains about 88% of cellulose and CipA8 was shown to bind to the substrate as efficiently as on Avicel, but an identically composed nonavalent synthetic complex failed to degrade significant amounts of this substrate (data not shown).

### Cloning and screening of cellulosomal proteins on softwood pulp

**Fig. 2:**
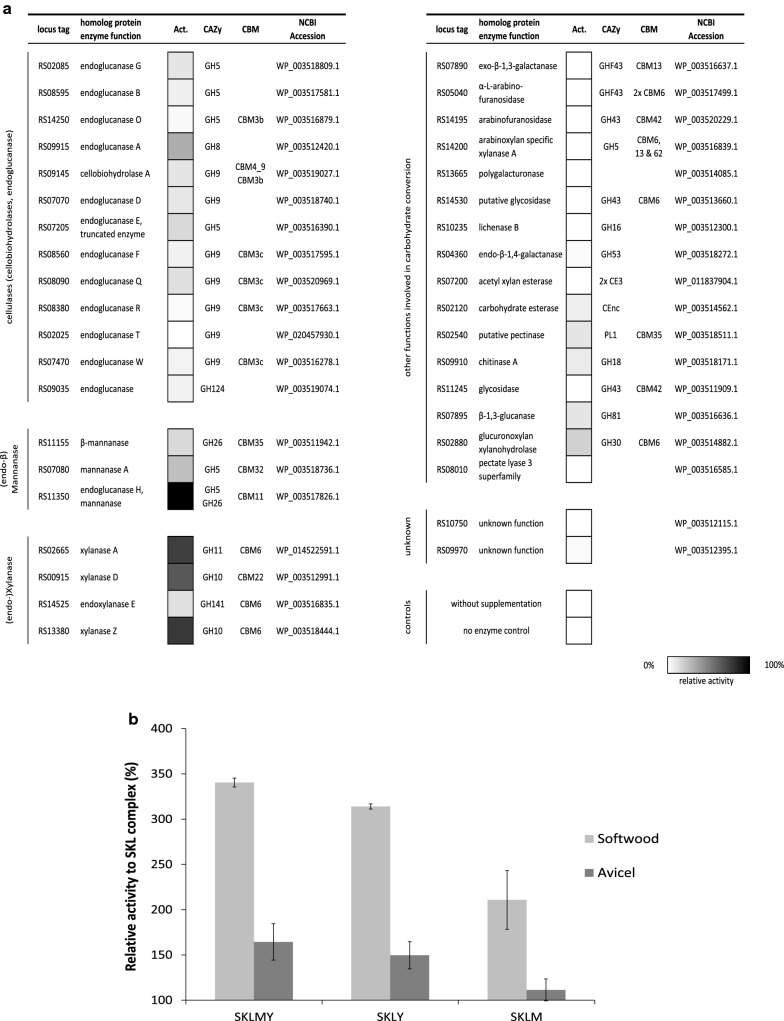
Screening of recombinantly expressed cellulosomal proteins on softwood. A minimized SKL complex was incubated with single-enzyme supplementations on 0.25% softwood, and soluble reducing sugars were measured after 2 days at 60 °C. **a** 38 proteins were supplemented to the SKL complex in identical molar stoichiometry and tested in duplicates. Activities are shown as heat map representations of reducing sugars released and quantified by DNS assay. Relative activity is depicted as follows: 100% relative activity equals black depiction, 0% no color. **b** A minimized SKL complex (relative activity = 100%) was incubated with single-enzyme combinations of Man26A and Xyn10Y. Soluble reducing sugars released from 0.25% (w/v) Avicel (dark gray) and softwood (light gray) were measured after 2 days at 60 °C. Each supplementation was added to the complex in identical stoichiometry (one supplement per cohesin). Data are shown as average values from at least duplicate (*n* = 2) measurements

In total, 73 dockerin type I-containing polypeptide sequences were predicted from in silico genome analysis of the *C. thermocellum* DSM 1313 genome and targeted for cloning and subsequent expression (Table 1). The proteins are either identical (100% sequence identity) or share very high amino acid sequence identity (99%) with the type strain *C. thermocellum* ATCC 27405 cellulosome components, of which many individual components are fully characterized. Furthermore, 16 dockerin-containing proteins were predicted that cannot directly be linked to carbohydrate hydrolysis (serpin, protease), contain only glycoside hydrolase (GH)-associated modules (fibronectin), have no predictable function at all (unknown modules) or are very small polypeptides only encoding dockerin type I modules (MW ≤ 15 kDa). We expressed 60 of the 73 polypeptides and purified them in soluble form, whereas 57 could be obtained as full-length protein only (see Additional file 2 summarizing all purified proteins). To analyze the impact of additional functions on complex activity, a stoichiometrically not fully loaded, minimized three component synthetic complex [SKL: Cel48S (Clo1313_2747), Cel9K (Clo1313_1809) and Cel5L (Clo1313_1816)] was mixed with 38 different single proteins and screened for higher substrate conversion efficiency (see “Methods” section for exact stoichiometries). After 2 days of incubation at 60 °C mainly (*endo*-)xylanase (Xyn11A: Clo1313_0521; Xyn10D: Clo1313_0177; Xyn10Z: Clo1313_2635) and β-mannanase functions (Man26A: Clo1313_2202; Man5A: Clo1313_1398; Cel5-26H: Clo1313_2234) were found to stimulate the complex activity on this substrate. The involved glycoside hydrolase families were GH10, GH11, GH5, and GH26, respectively (Fig. 2a).

**Table 1:** All cellulosomal proteins containing a dockerin type I module

Locus tag (Clo1313_)^a^	Alternative locus tag (CLO1313_)^b^	Homolog locus tag in type strain^c^	Homolog protein in type strain^c^	Protein sequence identity (%)^d^	Refs.	Domain structure (CAZy/Pfam)^e^	Molecular weight (kDa)	Expression and purification
2216	RS11245	Cthe_0015		100		CBM42, GH43	79	Soluble protein
2202	RS11155	Cthe_0032	Man26B	100		CBM35, GH26	67	Soluble protein
2189	RS11090	Cthe_0043	Cel9N	100	[23]	GH9, CBM3c	82	Soluble protein
2188	RS11085	Cthe_0044	CseP	100	[23]	CotH	62	Soluble protein
2122	RS10750	Cthe_0109		99		Un	12	Soluble protein
2043	RS10350	Cthe_0190	PinA	100	[24]	Fn3, serpin	68	Soluble protein
2042	RS10345	Cthe_0191	PinB	100	[24]	Fn3, serpin	68	Soluble protein
2022	RS10235	Cthe_0211	Lic16B	100	[25]	GH16	38	Soluble protein
1990	RS10065	Cthe_0239		99		LTD, LTD, Fn3, CotH	117	Not clonable
1983	RS10030	Cthe_0246		99		CBM35, PL11	89	Not clonable
1971	RS09970	Cthe_0258		100		(RCC1)_5_	51	Soluble protein
1960	RS09915	Cthe_0269	Cel8A	100	[26]	GH8	53	Soluble protein
1959	RS09910	Cthe_0270	Chi18A	100	[27]	GH18	55	Soluble protein
1955	RS09890	Cthe_0274	Cel9P	100	[10]	GH9	63	Soluble protein
1816	RS09185	Cthe_0405	Cel5L	100	[10]	GH5	58	Soluble protein
1809	Synthetic construct	Cthe_0412	Cel9K	100	[28]	GH9, Ig, CBM4_9, CBM3b	101	Soluble protein
1808	RS09145	Cthe_0413	Cbh9A	99	[29]	GH9, Ig, CBM4_9, CBM3b	138	Soluble protein
1788	RS09045	Cthe_0433	Lec9B	100	[10]	GH9, CBM3c	89	Soluble protein
1786	Synthetic construct	Cthe_0435	Cel124A	100	[30]	GH124	40	Soluble protein
1783	RS09020	Cthe_0438		100		Un	15	No purification
1701	RS08595	Cthe_0536	Cel5B	100	[31]	GH5	64	Soluble protein
1694	RS08560	Cthe_0543	Cel9F	100	[32]	GH9, CBM3c	82	Soluble protein
1659	RS08380	Cthe_0578	Cel9R	99	[33]	GH9, CBM3c	85	Soluble protein
1604	Synthetic construct	Cthe_0624	Cel9-44J	100	[34]	GH9, GH44, Ig, CBM4_9	178	Soluble protein
1603	RS08090	Cthe_0625	Cel9Q	100	[35]	GH9, CBM3c	80	Soluble protein
1587	RS08010	Cthe_0640		100		Pectate-lyase 3 superfamily	65	Soluble protein
1564	RS07895	Cthe_0660		99		GH81	86	Soluble protein
1563	RS07890	Cthe_0661	Gal43A	99	[36]	GH43, CBM13	64	Soluble protein
1494	RS07550	Cthe_0729		100		CBM	58	No expression
1477	RS07470	Cthe_0745	Cel9W	100	[10]	GH9, CBM3c	82	Soluble protein
1425	RS07205	Cthe_0797	Cel5E	99	[37]	GH5, CE2	90	Soluble protein
1425*	RS07205*	Cthe_0797*	tCel5E	100	[38]	GH5	54	Soluble protein
1424	RS07200	Cthe_0798	Ces3A	100	[39]	CE3, CE3	55	Soluble protein
1398	RS07080	Cthe_0821	Man5A	99	[40]	GH5, CBM32	60	Soluble protein
1396	RS07070	Cthe_0825	Cel9D	99	[41]	GH9, Ig	72	Soluble protein
1305	RS06630	Cthe_0912	Xyn10Y	100	[42]	CBM22, GH10, CBM22, CE1	120	Soluble protein
0987	RS05040	Cthe_1271		100		GH43, CBM6, CBM6	75	Soluble protein
0851	RS04370	Cthe_1398	Xgh74A	100	[43]	GH74	92	Soluble protein
0849	RS04360	Cthe_1400		100		GH53	47	Soluble protein
2234	RS11350	Cthe_1472	Cel5-26H	99	[44]	GH5, GH26, CBM11	102	Soluble protein
2479	RS12560	Cthe_1806		93		Un	236	Not clonable
2530	RS12825	Cthe_1838	Xyn10C	100	[45]	CBM22, GH10	70	Soluble protein
2564	RS13020	Cthe_1890		85		(LRR_5)_3_	76	Not clonable
2635	RS13380	Cthe_1963	Xyn10Z	99	[46]	CE1, CBM6, GH10	92	Soluble protein
2693	RS13665	Cthe_2038	Pgu28A	99		GH28 homology	92	Soluble protein
2747	Synthetic construct	Cthe_2089	Cel48S	100	[47]	GH48	83	Soluble protein
2793	RS14190	Cthe_2137		100		GH39, CBM35, CBM35	88	Insoluble protein
2794	RS14195	Cthe_2138		100		CBM42, GH43	66	Soluble protein
2795	RS14200	Cthe_2139		99		GH30, CBM42, GH43	111	Low expression yield
2805	RS14250	Cthe_2147	Cel5O	99	[48]	GH5, CBM3b	75	Soluble protein
2843	RS14430	Cthe_2179		98		PL1, CBM35, PL9	98	No expression
2856	RS14510	Cthe_2193	Xyl5A	99	[49]	GH5, CBM6, CBM13, CBM62	103	Soluble protein
2858	RS14520	Cthe_2194		96		CE1, CBM6	54	Insoluble protein
2859	RS14525	Cthe_2195	Xyn141E	99	[65]	GH141, CBM6	105	Soluble protein
2860	RS14530	Cthe_2196		100		GH43, CBM6	59	Soluble protein
2861	RS14535	Cthe_2197		74		GH2, CBM6	104	Truncated protein only
2944	RS14960	Cthe_2271		100		Un	19	No expression
3023	RS15380	Cthe_2360	Cel9U	99	[10]	GH9, CBM3b, CBM3c	105	Soluble protein
0135	RS00705	Cthe_2549		100		Un	37	Insoluble protein
0177	RS00915	Cthe_2590	Xyn10D	100	[10]	CBM22, GH10	72	Soluble protein, partially degraded
0349	RS01780	Cthe_2760	Cel9V	99	[10]	GH9, CBM3b, CBM3c	110	Soluble protein
0350	RS01785	Cthe_2761	Lec9A	99	[10]	GH9, CBM3c	80	Soluble protein
0399	RS02020	Cthe_2811	Man26A	100	[66]	CBM35, GH26	67	Soluble protein
0400	RS02025	Cthe_2812	Cel9T	100	[50]	GH9	69	Soluble protein
0413	RS02085	Cthe_2872	Cel5G	99	[51]	GH5	63	Soluble protein
0420	RS02120	Cthe_2879		99		CE-nc	55	Soluble protein, partially degraded
0500	RS02535	Cthe_2949		99		CE8	62	Soluble protein
0501	RS02540	Cthe_2950		99		PL1, CBM35	60	Soluble protein
0521	RS02665	Cthe_2972	Xyn11A	99	[52]	GH11, CBM6, CE4	74	Soluble protein
0563	RS02880	Cthe_3012		100		GH30, CBM6	71	Soluble protein
0685	RS03545	Cthe_3132		100		UN	47	Soluble/insoluble protein
0689	RS03565	Cthe_3136	CprA	100	[53]	Subtilisin-like serine protease	40	Insoluble protein
0693	RS03585	Cthe_3141		99		CE12, CBM35, CE12	91	Soluble protein

*GH* glycoside hydrolase family, *CBM* carbohydrate-binding module family, *Ig* glycoside hydrolase-associated immunoglobulin module, *CE* carbohydrate esterase family, *PL* polysaccharide lyase family, *UN* unknown module or module with unknown function, *LTD* lamin tail domain, *FN3* fibronectin module, *CotH* CotH spore coat protein kinase module, *RCC1* regulator of chromosome condensation, *LRR* leucin-rich repeat

^a^Gene feature record annotated as old locus tag for *C. thermocellum* DSM1313 in NCBI database (https://www.ncbi.nlm.nih.gov/nuccore/385777386)

^b^Current gene feature record annotated as locus tag in the NCBI database

^c^Homolog sequence annotation (locus tag and protein name) of type strain *C. thermocellum* ATCC 27405

^d^Sequence identity by blastP (https://blast.ncbi.nlm.nih.gov) against type strain *C. thermocellum* ATCC 27405 (% of protein sequence)

^e^Protein family classification based on carbohydrate-active enzyme (CAZy) database [54] (http://www.cazy.org) and Pfam database (http://pfam.sanger.ac.uk)

We raised the question, if an adapted complex would benefit from the addition of two additional functions, if these functions were synergistic, and if the selection of these additions would depend on the substrate composition. Based on the screening results (Fig. 2a) and the availability of recombinant cellulosomal proteins (Table 1), a set of component combinations was designed incorporating each enzymatic function necessary for the degradation of softwood: a reducing end and a non-reducing end exo-acting cellobiohydrolases (Cel48S and Cel9K), one cellobiose-releasing processive endoglucanase (Cel5L), whereby each cellulase is added in equimolar ratios and optionally the supplemental functions consisting of a mannanase function (Man26A, stochastically one cohesin per complex, equally to 12.5%) and a multi-modular xylanase (Xyn10Y, 12.5%), respectively (see “Methods” section for exact stoichiometric loading of each component). As a result, the presence of both enzymatic functions, the mannan-degrading (e.g. Man26A) and the xylanolytic (Xyn10Y) function (resulting in the SKLMY complex, Fig. 2b) led to a 3.5-fold increase in activity relative to the minimized SKL cellulase complex on 0.25% (w/v) of the substrate softwood; the addition of the multifunctional xylanase Xyn10Y alone had the highest impact (3.1× activity), followed by Man26A (2.1× activity). On microcrystalline cellulose the effect was far less pronounced, with only 65% higher activity of the SKLMY complex (Fig. 2b).

### Complex assembly and biochemical characterization of the optimized SKLMY complex

**Fig. 3:**
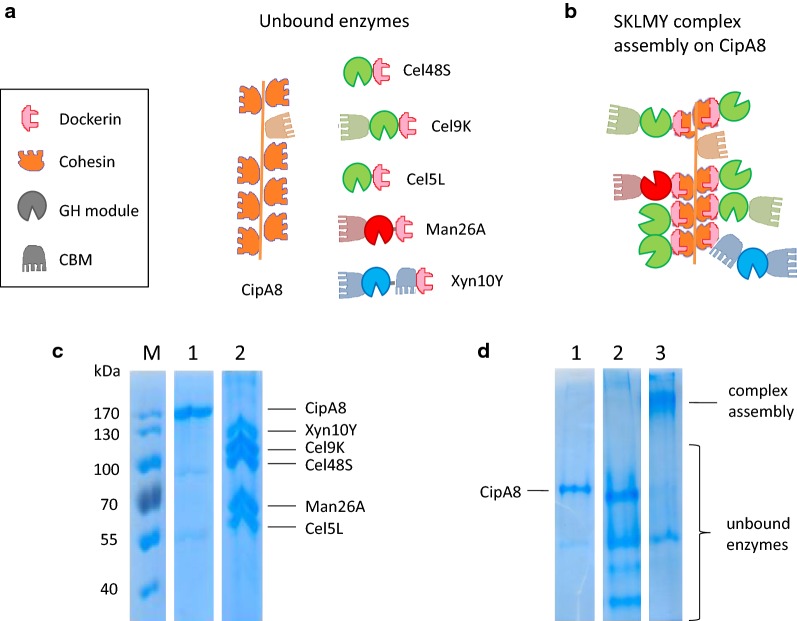
Assembly process of the SKLMY complex. **a** Schematic representation of the recombinant cellulosomal components Cel48S, Cel9K, Cel5L, Man26A and Xyn10Y, containing dockerin type 1-binding modules. The scaffoldin protein CipA8 comprises eight cohesin type I modules, enabling stoichiometric binding of eight dockerin-containing components via specific protein–protein interaction. **b** The assembly of the single components results in random combinations of macromolecular complexes, termed SKLMY. The order of components bound is arbitrary. **c** SDS-PAGE control of the assembly. CipA8 (3.8 µg in lane 1) and eight-time molar excess of single and unbound SKLMY components (15.3 µg loaded in lane 2) is mixed for the complex assembly reaction (19.1 µg in lane 3). **d** Native PAGE of single CipA8 (lane 1), unbound components (lane 2) and electrophoretic mobility up-shift upon SKLMY complex formation (lane 3)

The dockerin-containing single SKLMY components (Cel48S, Cel9K, Cel5L, Man26A, Xyn10Y) were assembled with the scaffoldin protein CipA8 via dockerin–cohesin interaction (schematic representation in Fig. 3a). Upon mixing the single enzymes in desired stoichiometric ratios (Cel48S: 25%, Cel9K: 25%, Cel5L: 25%, Man26A: 12.5%, Xyn10Y: 12.5%), binding occurs in a random fashion on the eight available cohesins of the CipA8 molecule. This approach is different to designer cellulosomes, where the order of the components is controlled by selectively binding each component to the corresponding binding module at a fixed position on the scaffoldin molecule [68, 69]. The approach of randomly assembling protein mixtures has been successfully applied for testing native and recombinant cellulosomal components from *C. thermocellum* [7], [13] and is assayed by complex purification using size-exclusion chromatography (see “Methods” section) and electrophoretic mobility shift analysis (EMSA) of the complexes visualized by native PAGE (Fig. 3c, d). After complexation, the five single enzymes and the scaffoldin protein were up-shifted indicating assemblage of the single components into a higher molecular weight protein complex.

**Fig. 4:**
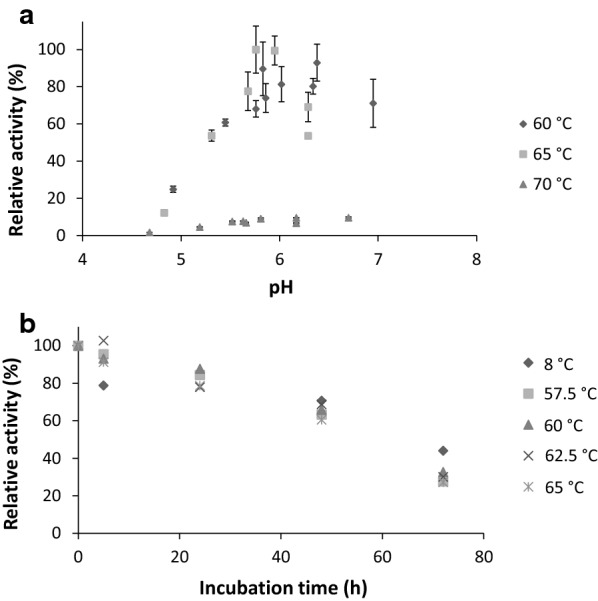
Biochemical properties of the optimized SKLMY enzyme complex on softwood pulp. **a** pH optima at three different temperatures around the temperature optimum of 60–65 °C after 36 h of incubation. **b** Thermo-inactivation kinetics of the SKLMY complex during incubation at different temperatures. 10 µg of complex was incubated on 0.25% (w/v) softwood and the concentration of liberated glucose was measured

The established pentavalent complex termed SKLMY was further characterized for enzyme kinetics and biochemical properties. All single enzymatic components (Cel48S, Cel9K, Cel5L, Man26A, Xyn10Y) were able to bind to all cohesin modules of CipA8. The synthetic SKLMY complex showed temperature and pH optima between 60 and 65 °C at around pH 5.8, whereas at higher temperature (70 °C) it was completely inactivated (Fig. 4a). The pentavalent synthetic complex displayed a high thermal stability over 2 days, retaining approximately 60–70% of its initial activity even at its temperature optimum. Noteworthy, inactivation of the complex strongly depended on the incubation time rather than the temperatures applied (Fig. 4b). The influence of common by-products such as purification agents (imidazole, ammonium sulfate) and cryo-preservatives (glycerol) was studied (Additional file 3). Imidazole is the most potent inhibitor of the recombinant cellulosome complex, which at concentrations as low as 5–10 mM resulted in a significant activity reduction (data not shown). Glycerol and ammonium sulfate above 10% saturation (w/v) (used for protein complex precipitation and purification) were also shown to be important inhibitors of hydrolysis that resulted in reduced activity (reduction by 25–50%). Sucrose used as another cryo-preservative showed comparable inhibition results (data not shown). The presence of bivalent metal ions (1 mM CoCl_2_, 1 mM MnCl_2_, 10 mM MgSO_4_) did not result in significant changes of the recombinant complex activity.

**Fig. 5:**
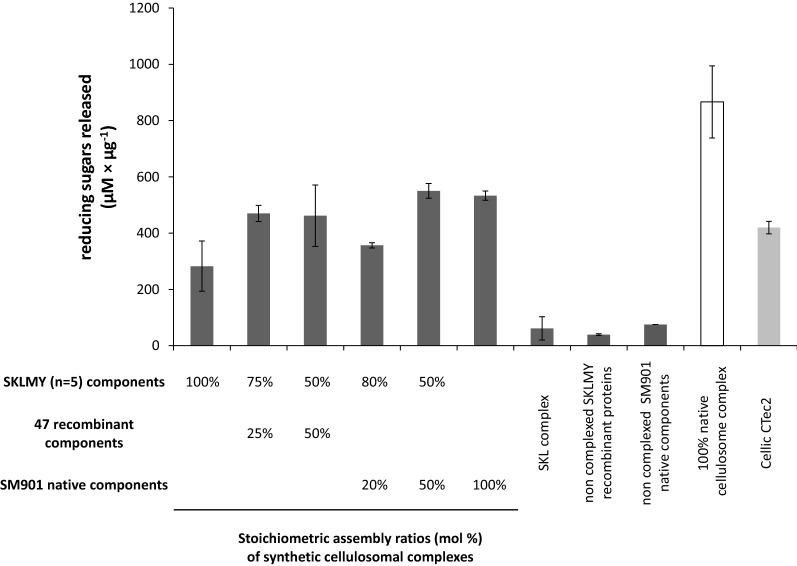
Comparison of commercial fungal cellulase with SKLMY complex on 0.25% (w/v) softwood pulp as substrate. The soluble fraction of reducing sugar ends was quantified after 2 days of incubation at optimal reaction conditions (60 °C for cellulosomal native and synthetic complexes at pH 5.8, and 50 °C at pH 5.0 for fungal enzyme preparation, respectively). The SKLMY complex was mixed with varying amounts (% of all cohesin-binding positions on CipA8) of native components (SM901 mutant extract) or an equimolar ratio of recombinant enzymes (*n* = 47, Additional file 4 for enzymatic composition). Enzyme loadings were as follows: Cellic CTec2, 7.6 µg per reaction; synthetic cellulosome complexes (each 1 µg); non-complexed enzyme control (− CipA: 13.2 µg); cellulosome complexes contain 3 µg of β-glucosidase as additive in the reaction mixture. Substrate loading was 1.25 mg per reaction. Bars represent average values ± standard deviation from three independent enzyme reactions

The enzyme efficiency of the established pentavalent SKLMY complex was assessed and compared with the fungal enzyme mixture Cellic CTec2 (Fig. 5). This complex supplemented with 47 additional recombinant enzymes (see Additional file 4 for the exact protein composition) or SM901 native free cellulosomal enzyme mixture showed comparable results with the existing commercial preparation (0.25% w/v softwood). The SKLMY complex showed approx. 30% of the activity of the native cellulosome on softwood, whereas supplementation of additional enzymes to the complex resulted in 50–60% of the overall activity of the native cellulosome.

## Discussion

Enzymatic degradation of cellulosic biomass is one of the most cost-intensive key reactions in the biomass-to-liquid process. Consequently, there is a huge demand in further optimization of cellulases. Advantageous properties are amongst other factors: (a) higher process temperature during hydrolysis reaction to avoid the contamination risk, (b) re-use of enzymes by resistance to thermally or chemically induced denaturation, (c) an enhanced hydrolytic efficiency through higher enzymatic activity, (d) reduced inhibition by high concentrations of oligo- or monosaccharides, (e) high yields of cellulases in their production and (f) adaptability of enzyme composition within the mixture depending on the substrate [8]. Fungal enzyme cocktails are regularly used today, as they can be economically produced in large amounts. They are still optimized further, for instance, by including accessory enzymes such as lytic polysaccharide monooxygenase (LPMOs). Advances have been made to optimize specific biochemical properties and by selecting special features when screening recombinant proteins, either by applying site-directed or random mutagenesis, exchange/deletion of peptide signatures and re-arrangement of functional modules, or by directed protein engineering and modeling [55, 56].

Although many sophisticated molecular biological tools are available for the engineering of fungal proteins [57], one major drawback in development is the labor-intensive adaptation of the enzyme composition when optimizing for different substrates (depending on the polysaccharide composition, pre-treatment conditions, amongst others). As another implication, each alteration of the cellulase system may again interfere with complementary/synergistic enzymatic functions of the whole enzyme mixture. Thereby the complexity of the polysaccharide matrices dictates the complexity of the cellulase formulation and accessory enzymes.

Saccharolytic clostridia are known to produce a battery of extracellular glycoside hydrolases to degrade a diversity of polysaccharides [58]. Hemicelluloses are depolymerized by a diverse set of xylan-, mannan-, arabinogalactan-, xyloglucan-, and pectin-degrading enzymes. Side chains and oligosaccharides can be further depolymerized by the action of arabinofuranosidases and β-xylosidases. The native cellulosomal enzyme complex from *C. thermocellum*, containing all of these functions in addition to numerous cellulases, is regarded as one of the most efficient cellulose-degrading systems known to date. Since the first description of this supra-molecular complex in 1983 [3], it became clear that its industrial use is mainly hampered by low production yield from the anaerobic *C. thermocellum* cultures, and the immense complexity of over 70 known components.

The adaptation of the cellulosome enzymatic composition strongly depends on the nature of the substrate to be degraded, as was shown by transcriptomic and proteomic data [10, 14, 59, 60]. Consequently, we reasoned that a fully synthetic cellulosome complex would allow for the fast adaptation on a substrate while reducing the number of enzymatic components when single enzymes were expressed and added separately. Further advantages are the higher temperature stability of the cellulosomal proteins with optimal incubation temperatures around 60–65 °C compared to 50 °C of most fungal enzymes.

By attempting to express and purify all genetically encoded cellulosomal components of *C. thermocellum*, 57 of all 73 dockerin-containing components (78%) could be obtained in full length and soluble form. The reasons for the failure to obtain 16 of the components recombinantly are manifold and maybe connected to the limitations of heterologous protein expression encoded by the genomic inserts [61–63]. In some cases, enhanced expression levels were only observed with special *E. coli* expression systems that function via co-expression of cold-adapted chaperonins using Arctic Express (for Clo1313_2747 and Clo1313_2859) or via overcoming tRNA pool depletion by the co-expression of genes for rare tRNAs using BL21 Codon Plus (for Clo1313_2858 and Clo1313_0685) or Rosetta-gami B (for Clo1313_2795 and Clo1313_0501). Three proteins (Clo1313_0177, Clo1313_0420) and Clo1313_2861) could only be obtained in truncated or mixed forms, most likely due to proteolytic cleavage at flexible and exposed linkers between two protein modules.

Starting from a stoichiometrically not fully loaded and minimized cellulase complex (SKL), we supplemented single enzymes to study their effect on the complex activity. Interestingly, we were able to identify seven single cellulosomal enzymes belonging to two major functional groups which we regard as the core enzymatic requirements:

The activity-boosting enzymes (Man5A, Man26A, Man26B, Cel5-26H) are known for activity on mannans (β-mannanase, exo-β-1,4-mannobiohydrolase; β-1,3-xylanase (EC 3.2.1.32); lichenase/*endo*-β-1,3-1,4-glucanase; mannobiose-producing exo-β-mannanase). Especially galactomannans are highly abundant in softwoods (20-25% of the dry mass), whereby its backbone consists of β-(1,4)-linked β-d-glucopyranose and β-d-mannopyranose residues. Further acetyl groups and α-(1,6)-d-galactopyranose are present as partial substituents [64]. As a second enhancing group, xylanases of the families GH10 and GH11 (Xyn11A, Xyn10C, Xyn10D, Xyn10Y, Xyn10Z) and the xylanolytic enzyme Xyn141E with high sequence similarity to the recently described family GH141 [65] could be identified. These enzymes hydrolyze xylans, highly abundant and variable hemicelluloses in nature that share a backbone of β-(1,4)-d-xylose units with diverse substitutions.

Due to these results, we reasoned that these two general accessory functions, mannanolytic and xylanolytic activities, are needed to boost the activity of the basal cellulase complex for this specific substrate. Verification of this assumption was possible by assembling in vitro combinations of complex compositions. By incorporating only two additional enzymes, Xyn10Y and Man26A, an approximately threefold higher enzymatic complex activity could be obtained. We suggest that the pentavalent SKLMY complex must contain a minimum number of five single components (with at least five distinct functions, respectively) bound on the carrier protein CipA8 to effectively hydrolyze softwood pulp as the substrate: one processive endoglucanase producing cellobiose as main hydrolysis product (Cel5L), two cellobiohydrolases (CBHs, Cel48S and Cel9K) with specificity from the reducing and non-reducing end of the polysaccharide chain, respectively, a mannanase of the GH26 family (Man26A, [66]) and a xylan-specific multifunctional feruloyl-esterase containing xylanase (Xyn10Y [67]). In addition, β-glucosidase was added to relieve the inhibitory effect of cellobiose on the CBH enzymes. Other studies also focused on the incorporation of single xylanolytic functions into designer cellulosomes for higher enzymatic efficiencies, as successfully shown for xylanases of *Thermobifida fusca* on wheat straw [68, 69]. Noteworthy, the complex optimization is not finished at this stage of complex development. Another initial complex combination than SKL may result in additional synergies that might have been missed in our approach. By establishing the synthetic complex containing almost all residual recombinant enzymes, a significant boost in enzyme activity was observed in our study. This in turn may be due to hidden enzyme synergies that we could not yet uncover. Due to the indefinite number of possible enzyme and stoichiometric combinations, more advanced, automated and high-throughput screening approaches will have to be applied.

Our minimalized but fully synthetic enzyme mix achieved almost 60% of the activity of the commercially available fungal cellulase blend Cellic CTec2 on softwood pulp as the substrate. Further addition of over 40 fully synthetic components of known and unknown functions, or alternatively the native protein mixture SM901 purified from a CipA-deficient mutant (from mutant SM1, [17]) led to enzymatic efficiencies comparable to a commercial fungal cellulase preparation. An identical enzyme complex composition did not result in comparable hydrolytic efficiency when tested on other cellulosic substrates (e.g. Avicel), as the SKLMY complex was optimized for softwood pulp degradation. Depending on the substrate constituents, further complex optimization will be necessary as important enzymatic functions may still be missing. To the very best of our knowledge, the superior hydrolytic efficiency of the cellulosome on more complex substrates and under process-relevant conditions has still not been proven so far, but should be reachable in the near future by engineering synthetic cellulosome analogs or designer cellulosomes.

An important aspect that could not be addressed within this study was the role of the stoichiometric loading of diverse enzymatic functions and ratios between components within the cellulosomal multi-enzyme complex. Numerous studies tried to answer this question by employing transcriptomic and proteomic analyses to understand the complex adaptation on different substrates. However, recent results indicate that the enzymatic complexity of the cellulosome is a key feature for its high hydrolytic efficiency on cellulosic substrates [13, 14, 16]. Furthermore, this principle seems to hold true also for other cellulosomal multi-enzyme systems from other cellulolytic bacteria such as *Acetivibrio cellulolyticus* and *Ruminococcus flavefaciens* [70, 71]. As a consequence, a high-throughput screening strategy is needed to understand the interplay between single enzymatic activities and synergies between the functional groups and proximity of single components, and to build up computational models. This knowledge may help to predict and adapt fully synthetic complexes to virtually any kind of polysaccharide from plant-derived biomass, in dependence of the substrate composition requirements.

## Conclusions

Inspired by the supra-modular extracellular cellulase complex from *C. thermocellum*, we designed fully synthetic cellulosome complexes for enhanced degradation of softwood pulp as cellulose-based substrate. To this end, we expressed and purified 60 single enzymatic components to systematically study the core enzymatic modalities needed to hydrolyze softwood pulp. Two major function classes, xylanase and mannanase enzymes, were incorporated into a pentavalent recombinant cellulase complex that was characterized biochemically. In direct comparison, the enzymatic efficiency of a fully synthetic cellulosome is, even without stoichiometric optimization, comparable with the commercial fungal enzyme cocktail Cellic CTec2. This study underscores the prospect to use synthetic cellulosome complexes for a fast and versatile adaptation of single enzymatic functions to achieve high activity on cellulosic substrates.

## Additional files

**Additional file 1.** Oligonucleotides used in this study.

**Additional file 2.** SDS-PAGE summary of all recombinantly expressed dockerin type I containing proteins used in this study (60 different components of *C. thermocellum* cellulosome). Each protein is shown after the purification process, including the final heat precipitation step. Enzyme names and Clo1313 numbers are shown above the corresponding SDS-PAGE picture. Molecular weight standards are depicted in kDa. Proteins marked with asterisks are truncated versions of the original protein.

**Additional file 3.** Effect of inhibitors on the cellulosomalcomplexes. The glucose concentration was measured using the Glu-HK determination kit (Megazyme). The residual complex activity was assessed using 0.5 mL standard reaction mixture containing 0.25% (w/v) of the substrate for one to 2 days of incubation at 60 °C. The initial activity at time point 0 corresponds to 100% relative activity.

**Additional file 4.** Overview of 47 recombinant proteins. All proteins were mixed in equimolar amounts before adding them to the pentavalent SKLMY complex.

## Abbreviations

CBH: cellobiohydrolase
CBM: carbohydrate-binding module
CE: carbohydrate esterase
CotH: CotH spore coat protein kinase module
EMSA: electrophoretic mobility shift assay
FN3: fibronectin module
GH: glycoside hydrolase
Ig: glycoside hydrolase-associated immunoglobulin module
IPTG: isopropyl-β-d-thiogalactopyranoside
LPMO: lytic polysaccharide monooxygenase
LRR: leucin-rich repeat
LTD: lamin tail domain
PL: polysaccharide lyase
RCC: regulator of chromosome condensation
RT: room temperature
UN: unknown module or module with unknown function
