# Supplementary material for: Optimizing the composition of a synthetic cellulosome complex for the hydrolysis of softwood pulp: identification of the enzymatic core functions and biochemical complex characterization
Source: Biotechnol Biofuels. 2018 Aug 9;11:220. doi: 10.1186/s13068-018-1220-y (PMC6083626; doi:10.1186/s13068-018-1220-y)
Supplement: Supplementary file 1 — Additional file 1. Oligonucleotides used in this study. [file 13068_2018_1220_MOESM1_ESM.docx]

**Additional file S1: Oligonucleotides used in this study.**

| **primer name (_locus tag ID)** | **sequence (5' to 3')** |
| --- | --- |
| Clo1313_0135-f | TTAAGAAGGAGATATACATATGGCCTATCTGGATAATGAGCTGT |
| Clo1313_0135-r | TCAGTGGTGGTGGTGGTGGTGCTCGAGTTTCTTGTTTGCAGGGAAGTCT |
| Clo1313_0177-f | GATCGAGCTCGAAGGCAATTTACTTTTCAACC |
| Clo1313_0177-r | GATCCTCGAGACGTTTTAAAGGCAATTCATCTA |
| Clo1313_0349-f | GATCGAGCTCGCACCACCTGCGACTTTTAC |
| Clo1313_0349-r | GATCCTCGAGAAAAGACGTTATTATGCCAAGAA |
| Clo1313_0350-f | GATCGGATCCGAGCCAAAATTTAACTATGTAGA |
| Clo1313_0350-r | GATCCTCGAGCTGCTCTACGGGGAACTTAT |
| Clo1313_0399-f | TTAAGAAGGAGATATACATATGCTTTCTGACGGGGATAAGTATG |
| Clo1313_0399-r | TCAGTGGTGGTGGTGGTGGTGCTCGAGCTGTTGTTCAACGGGAAAACTC |
| Clo1313_0400-f | GATCGGATCCGAATACAATTATGCAAAGGCGC |
| Clo1313_0400-r | GATCCTCGAGTATAGGGAGAGACGGTATGC |
| Clo1313_0413-f | GATCGGATCCAATACCGGTTCAACAGCTACG |
| Clo1313_0413-r | GATCCTCGAGGGTGGTGTGCGGCAGTTTG |
| Clo1313_0420-f | TTAAGAAGGAGATATACATATGAATGGCGGAAAATTGGGAGACA |
| Clo1313_0420-r | TCAGTGGTGGTGGTGGTGGTGCTCGAGATTAACGGGCATTTCAAAAGAT |
| Clo1313_0500-f | TTAAGAAGGAGATATACATATGGTCAATGCGGATATAATAGTTG |
| Clo1313_0500-r | TCAGTGGTGGTGGTGGTGGTGCTCGAGTACAGGCAATTGTGTAATTGTT |
| Clo1313_0501-f | TTAAGAAGGAGATATACATATGACCGTGTCACCTTTGGATAGAC |
| Clo1313_0501-r | TCAGTGGTGGTGGTGGTGGTGCTCGAGATTTCTCCTTATTTCGAGTTTA |
| Clo1313_0521-f | GATCCATATGGTGGTAATTACGTCAAACCAG |
| Clo1313_0521-r | GATCCTCGAGCGGTACAGAGTTATACATTCTT |
| Clo1313_0563-f | TTAAGAAGGAGATATACATATGGAAACGGCAACAATCAACTTGT |
| Clo1313_0563-r | TCAGTGGTGGTGGTGGTGGTGCTCGAGTGATTCTACAGGAAATTTATCA |
| Clo1313_0685-f | TTAAGAAGGAGATATACATATGGAGAGCGTACTGCAAGACAGGA |
| Clo1313_0685-r | TCAGTGGTGGTGGTGGTGGTGCTCGAGTTTTTCAACAGGAAATATATCT |
| Clo1313_0689-f | TTAAGAAGGAGATATACATATGGACATAGGAGACCATGGCACAC |
| Clo1313_0689-r | TCAGTGGTGGTGGTGGTGGTGCTCGAGTTGATTTCCGACAGGTAGATGA |
| Clo1313_0693-f | TTAAGAAGGAGATATACATATGTTGCGTATCAGTGCAGCCCCGA |
| Clo1313_0693-r | GGTGGTGGTGGTGGTGCTCGAGATTGGTTTTCAAATACTTCGCA |
| Clo1313_0849-f | TTAAGAAGGAGATATACATATGGCGGCTCCAACTTTTGCAAAGG |
| Clo1313_0849-r | TCAGTGGTGGTGGTGGTGGTGCTCGAGTTTTACAGGAAGTGATGGTATG |
| Clo1313_0851-f | TTAAGAAGGAGATATACATATGACCAGCGTGCCTTACAAATGGG |
| Clo1313_0851-r | TCAGTGGTGGTGGTGGTGGTGCTCGAGTTTTTTCGGCAGCTCCGGAATG |
| Clo1313_0851-f | TTAAGAAGGAGATATACATATGGATGACATTTATCCGGGACTTA |
| Clo1313_0851-r | TCAGTGGTGGTGGTGGTGGTGCTCGAGTGGTGTCACTATTCTCGTATCC |
| Clo1313_0987-f | TTTAAGAAGGAGATATACATATGTATCCGATTTTCTCACAGCGT |
| Clo1313_0987-r | TCAGTGGTGGTGGTGGTGGTGCTCGAGTATCGGGAAATTTTCTATTTCC |
| Clo1313_1305-f | TTAAGAAGGAGATATACATATGTATGAAGTGGTTCATGACACTT |
| Clo1313_1305-r | TCAGTGGTGGTGGTGGTGGTGCTCGAGTTCATGGAAGAAATATGGAAGT |
| Clo1313_1396-f | GATCGGATCCGAGACCAAAGTGTCAGCTGC |
| Clo1313_1396-r | GATCCTCGAGTATTGGTAATTTCTCGATTACCC |
| Clo1313_1424-f | TTAAGAAGGAGATATACATATGCCGGCTGCGTCAAAAACGATAA |
| Clo1313_1424-r | TCAGTGGTGGTGGTGGTGGTGCTCGAGATTTCTAAGAAGTATTCTCTTT |
| Clo1313_1425-f | TGAAGACAATCCGGGCATTTTGTATAACGGAAGAT |
| Clo1313_1425-r | CTCAGTGGTGGTGGTGGTGGTGCTCGAGCCATCCAAGCTTGTTTTTTATTTCCGC |
| Clo1313_1425-f | GATCGGATCCTCACCGGTAAAAGGCTTTCAG |
| Clo1313_1425-r | GATCCTCGAGGCCCGGATTGTCTTCATCAG |
| Clo1313_1477-f | GATCGGATCCACTACATTCAACTACGGAGAAG |
| Clo1313_1477-r | GATCCTCGAGAGGTGCGTAAGGCAGTTTGC |
| Clo1313_1494-f | TTAAGAAGGAGATATACATATGGAGCCACCACCGATATATGGCG |
| Clo1313_1494-r | TCAGTGGTGGTGGTGGTGGTGCTCGAGCCATCCTGGGCCGCCTGGGAAC |
| Clo1313_1563-f | TTAAGAAGGAGATATACATATGGAAGGGGTTATAGTCAACGGAA |
| Clo1313_1563-r | TCAGTGGTGGTGGTGGTGGTGCTCGAGCAAATCCACTTCCATAAGCAAG |
| Clo1313_1564-f | TTAAGAAGGAGATATACATATGCCACCGGGTGCTAAGGTACCTC |
| Clo1313_1564-r | TCAGTGGTGGTGGTGGTGGTGCTCGAGAAGCGGATAATTTCTCAATATCA |
| Clo1313_1587-f | TTAAGAAGGAGATATACATATGGAAATAAACGGTGAAGTTATTG |
| Clo1313_1587-r | TCAGTGGTGGTGGTGGTGGTGCTCGAGACTCTTTTCGGCAGGAAACTTA |
| Clo1313_1603-f | GATCAAGCTTTGCTGACGGCATTTATTCTTCC |
| Clo1313_1603-r | GATCCTCGAGTACCGGAAATTTATCTATTATACGG |
| Clo1313_1604-f | AAGAAGGAGATATACATATGACAGTTGCTCCTGAAGGCTACAGG |
| Clo1313_1604-r | TCAGTGGTGGTGGTGGTGGTGCTCGAGCCAGTCAATAGCATCTACATAG |
| Clo1313_1659-f | GATCGGATCCGACTATAACTATGGAGAAGCAC |
| Clo1313_1659-r | GATCCTCGAGTGAATTTCCGGGTATGGTTGG |
| Clo1313_1694-f | GATCGAGCTCGATTTCAACTATGGTGAGGCA |
| Clo1313_1694-r | GATCCTCGAGCTGTTCAGCCGGGAATTTTTC |
| Clo1313_1701-f | GATCGGATCCGAAGGGTCATATGCTGATTTG |
| Clo1313_1701-r | GATCCTCGAGTTTATACGGCAACTCACTTATG |
| Clo1313_1783-f | TTAAGAAGGAGATATACATATGGTGGCTAATACTGCAGATACAA |
| Clo1313_1783-r | TCAGTGGTGGTGGTGGTGGTGCTCGAGTTTCAAGTAGCTTAAATCAATC |
| Clo1313_1786-f | TTAAGAAGGAGATATACATATGAATAAGGCAGTTATTGGAGATG |
| Clo1313_1786-r | TCAGTGGTGGTGGTGGTGGTGCTCGAGGTAATAAATCTCCCATGGATTG |
| Clo1313_1788-f | GATCGAGCTCAATGTGGAATACAACTATGCAAA |
| Clo1313_1788-r | GATCCTCGAGTTTTATCGGAATAACCTCGATTG |
| Clo1313_1808-f | GATCGAGCTCGAAGATAATTCTTCGACTTTGC |
| Clo1313_1808-r | GATCCTCGAGTCGATATGGCAATTCTTCTATG |
| Clo1313_1816-f | GATCGGATCCGATCCGAACAATGACGACTG |
| Clo1313_1816-r | GATCCTCGAGTATTGGTATTTTAAGCACTTTCC |
| Clo1313_1955-f | GATCGGATCCGATTATGCCACCGCATTAAAAT |
| Clo1313_1955-r | GATCCTCGAGATTTATGATGTTTCCATAGATATC |
| Clo1313_1959-f | TTAAGAAGGAGATATACATATGGACGACTCTCTTCCGACAAAAA |
| Clo1313_1959-r | TCAGTGGTGGTGGTGGTGGTGCTCGAGATCATCAACAGGTATATTGTCA |
| Clo1313_1960-f | AGCGGCAGGTGAGCTCTTTAA |
| Clo1313_1960-r | GTAGGTGGTCGACGCTCTTTAT |
| Clo1313_1971-f | TTAAGAAGGAGATATACATATGGACGGTAAAAATGTGGTTTTAG |
| Clo1313_1971-r | TCAGTGGTGGTGGTGGTGGTGCTCGAGTTTTCTTTCAAATACTTTTACG |
| Clo1313_1983-f | TTTAAGAAGGAGATATACATATGGGACCGGCTTCAACCAAGTAT |
| Clo1313_1983-r | TCAGTGGTGGTGGTGGTGGTGCTCGAGCGGCACAAGGTAAATATTTGGC |
| Clo1313_1990-f | TTAAGAAGGAGATATACATATGGCGAGCCAGACCCTTTTTATTA |
| Clo1313_1990-r | TCAGTGGTGGTGGTGGTGGTGCTCGAGCTTCAACTGTACCGGGAACTTA |
| Clo1313_2022-f | TTAAGAAGGAGATATACATATGGCCGCAACTGTGGTAAATACGC |
| Clo1313_2022-r | TCAGTGGTGGTGGTGGTGGTGCTCGAGAAGTGACGGAATTGCCCGTATCA |
| Clo1313_2042-f | TTAAGAAGGAGATATACATATGAATTGGTACACTTATTACGAGT |
| Clo1313_2042-r | TCAGTGGTGGTGGTGGTGGTGCTCGAGATTAATCAACTCACCTT |
| Clo1313_2043-f | TTAAGAAGGAGATATACATATGGGAAACTGGAAAACTTATTACG |
| Clo1313_2043-r | TCAGTGGTGGTGGTGGTGGTGCTCGAGATTAATCAACCCGCCGTCACAT |
| Clo1313_2122-f | TTAAGAAGGAGATATACATATGGATGTACCATTTTCCGGCAAAT |
| Clo1313_2122-r | TCAGTGGTGGTGGTGGTGGTGCTCGAGATCTATACGATAATCGCCAATC |
| Clo1313_2188-f | TTAAGAAGGAGATATACATATGTCCAGACCTGAGGGCTGGACGG |
| Clo1313_2188-r | TCAGTGGTGGTGGTGGTGGTGCTCGAGTAAGGATGGAATGGCTTTTGCA |
| Clo1313_2189-f | GATCGAGCTCTCTTCGCCTCGTTACGGCG |
| Clo1313_2189-r | GATCCTCGAGTATAGGTAACGAACCAATTAACT |
| Clo1313_2202-f | TTAAGAAGGAGATATACATATGTATTCCCTTCCTGTGGACGTTG |
| Clo1313_2202-r | TCAGTGGTGGTGGTGGTGGTGCTCGAGCTTTTGTGTAACGGGAAATTTT |
| Clo1313_2216-f | TTAAGAAGGAGATATACATATGAACCCGATAACAAAAGCAAAAT |
| Clo1313_2216-r | TCAGTGGTGGTGGTGGTGGTGCTCGAGCAGTTGGCTGCCCCAAATTGTG |
| Clo1313_2234-f | GATCGGATCCTACAACAGTGGTTTAAAAATCGG |
| Clo1313_2234-r | GATCCTCGAGTATGGGTATTTCACTGATGGC |
| Clo1313_2479-f | TTAAGAAGGAGATATACATATGAATGCTTTTGAAGATCCATTTG |
| Clo1313_2479-r | TCAGTGGTGGTGGTGGTGGTGCTCGAGATAGCGGGAAGGTAATGCGTTA |
| Clo1313_2530-f | TTAAGAAGGAGATATACATATGGCTCTGATTTACGATGATTTTG |
| Clo1313_2530-r | TCAGTGGTGGTGGTGGTGGTGCTCGAGAAGTTCTCTCAGAACGAGTTTT |
| Clo1313_2564-f | TTAAGAAGGAGATATACATATGACATTATACGGTGACTTAAATG |
| Clo1313_2564-r | TCAGTGGTGGTGGTGGTGGTGCTCGAGTTTCCAACCTTTTGGAATATCA |
| Clo1313_2635-f | GATCGGATCCTCCTTGCCAACCATGCCGC |
| Clo1313_2635-r | GATCCTCGAGATAGCCCATAAGAGCTTCCTT |
| Clo1313_2693-f | TTAAGAAGGAGATATACATATGGATGGTGGACAGCTTATTATCT |
| Clo1313_2693-r | TCAGTGGTGGTGGTGGTGGTGCTCGAGTTGCGGAAAAGCATCAATAATT |
| Clo1313_2793-f | TTAAGAAGGAGATATACATATGGGGGAAATTTGTTATGGTGCCA |
| Clo1313_2793-r | TCAGTGGTGGTGGTGGTGGTGCTCGAGGTTAGTTTCTGCCGGGAATGCT |
| Clo1313_2794-f | TTAAGAAGGAGATATACATATGTCAACGGGTGCCGATGGTGCTA |
| Clo1313_2794-r | TCAGTGGTGGTGGTGGTGGTGCTCGAGTGTCTGATTTACCCAGGGCGGT |
| Clo1313_2795-f | TTAAGAAGGAGATATACATATGGCAACAACAATTACCATAGACC |
| Clo1313_2795-r | TCAGTGGTGGTGGTGGTGGTGCTCGAGAACCGGGAAAGAATCTATGCTG |
| Clo1313_2805-f | GATCGGATCCGACACTTCTGAAGAACCCGC |
| Clo1313_2805-r | GATCCTCGAGTTCATTTTGGTTTTCTTCCACC |
| Clo1313_2843-f | TTAAGAAGGAGATATACATATGCCAAGCTTTGAACTGGTTGGAT |
| Clo1313_2843-r | TCAGTGGTGGTGGTGGTGGTGCTCGAGTTTAAATATTGCATTGTCAAGT |
| Clo1313_2856-f | TTAAGAAGGAGATATACATATGCCGCAACGTGGCCGGCCGCGTC |
| Clo1313_2856-r | TCAGTGGTGGTGGTGGTGGTGCTCGAGTAAAGTAGTTATGACACGGAGT |
| Clo1313_2858-f | TTAAGAAGGAGATATACATATGTCGCTGCCAACTATGCCGCCGT |
| Clo1313_2858-r | TCAGTGGTGGTGGTGGTGGTGCTCGAGAAAATTCGTTACAAACCGAAGT |
| Clo1313_2859-f | TTAAGAAGGAGATATACATATGACTACATGGAATGAAAATATTG |
| Clo1313_2859-r | TCAGTGGTGGTGGTGGTGGTGCTCGAGAAAAGTAGGTATAATTTGAAGC |
| Clo1313_2860-f | TTAAGAAGGAGATATACATATGAATCCGATAGTACAAACAATCT |
| Clo1313_2860-r | TCAGTGGTGGTGGTGGTGGTGCTCGAGATTTTGAGGAAAATCCGATATA |
| Clo1313_2861-f | TTAAGAAGGAGATATACATATGATGAACAGCCAGGTATGGATAA |
| Clo1313_2861-r | TCAGTGGTGGTGGTGGTGGTGCTCGAGAGACCGAGCATAATCATCAAAA |
| Clo1313_2944-f | TTAAGAAGGAGATATACATATGCATCAATGCAGTGTGACGGTAA |
| Clo1313_2944-r | TCAGTGGTGGTGGTGGTGGTGCTCGAGGCTTTGCGGACATACCGGGAAA |
| Clo1313_3023-f | GATCGAGCTCGCCGAACCGGAATATAATTTTG |
| Clo1313_3023-r | GATCCTCGAGCTTTACGGGCAGTTTTTCTATT |
| Cthe_0071-f | GATCGGATCCCTGATAATCACAATCAAAAACAG |
| Cthe_0071-r | GATCAAGCTTGCCTACGGTGTTATTACCTG |
| Q60026_THEBR-f | AAGAAGGAGATATACATATGGCAAAATTTCCAAGAGATTTCG |
| Q60026_THEBR-r | CTCGAGTGCGGCCGCAAGATCTTCGATACCATCATCCAAT |
| TT_P0042-f | GATCGAATTCATGACCGAGAACGCCGAAAA |
| TT_P0042-r | GATCAAGCTTGGTCTGGGCCCGCGCG |
|  |  |
|  |  |
