# Supplementary material for: Optimizing the composition of a synthetic cellulosome complex for the hydrolysis of softwood pulp: identification of the enzymatic core functions and biochemical complex characterization
Source: Biotechnol Biofuels. 2018 Aug 9;11:220. doi: 10.1186/s13068-018-1220-y (PMC6083626; doi:10.1186/s13068-018-1220-y)

Additional file S2: SDS-PAGE summary of all recombinantly expressed dockerin-type I containing proteins used in this study (60 different components of *C. thermocellum* cellulosome). Each protein is shown after the purification process, including the final heat precipitation step. Enzyme names and Clo1313 numbers are shown above the corresponding SDS-PAGE picture. Molecular weight standards are depicted in kDa. Proteins marked with asterisks are truncated versions of the original protein.

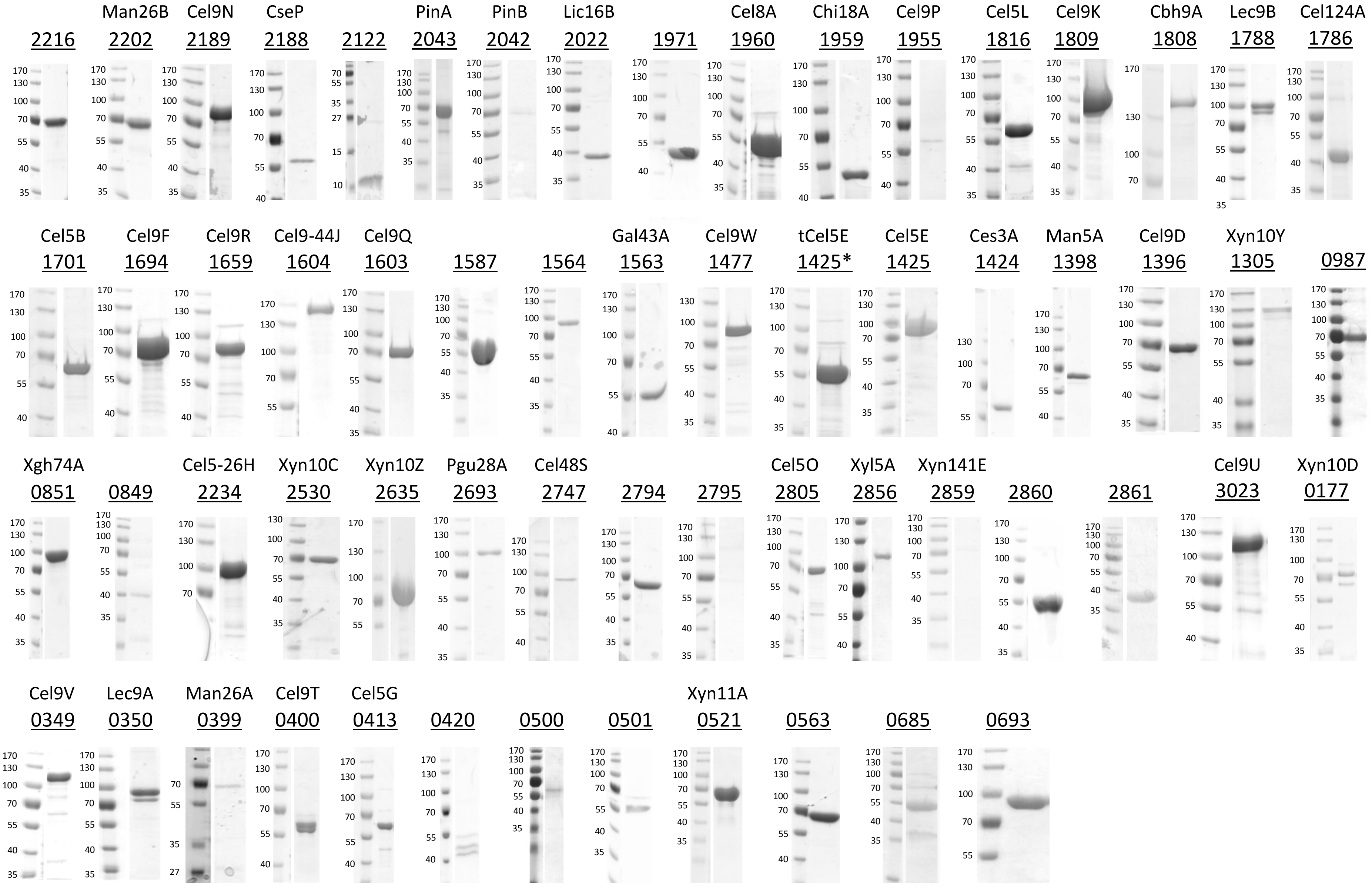

Supplement: Supplementary file 2 — Additional file 2. SDS-PAGE summary of all recombinantly expressed dockerin type I containing proteins used in this study (60 different components of C. thermocellum cellulosome). Each protein is shown after the purification process, including the final heat precipitation step. Enzyme names and Clo1313 numbers are shown above the corresponding SDS-PAGE picture. Molecular weight standards are depicted in kDa. Proteins marked with asterisks are truncated versions of the original protein. [file 13068_2018_1220_MOESM2_ESM.pdf]
