# Supplementary material for: Optimizing the composition of a synthetic cellulosome complex for the hydrolysis of softwood pulp: identification of the enzymatic core functions and biochemical complex characterization
Source: Biotechnol Biofuels. 2018 Aug 9;11:220. doi: 10.1186/s13068-018-1220-y (PMC6083626; doi:10.1186/s13068-018-1220-y)

Additional file S3: Effect of inhibitors on the cellulosomal complexes. The glucose concentration was measured using the Glu-HK determination kit (Megazyme). The residual complex activity was assessed using 0.5 mL standard reaction mixture containing 0.25 % (w/v) of the substrate for one to two days of incubation at 60 °C. The initial activity at time point 0 corresponds to 100 % relative activity.

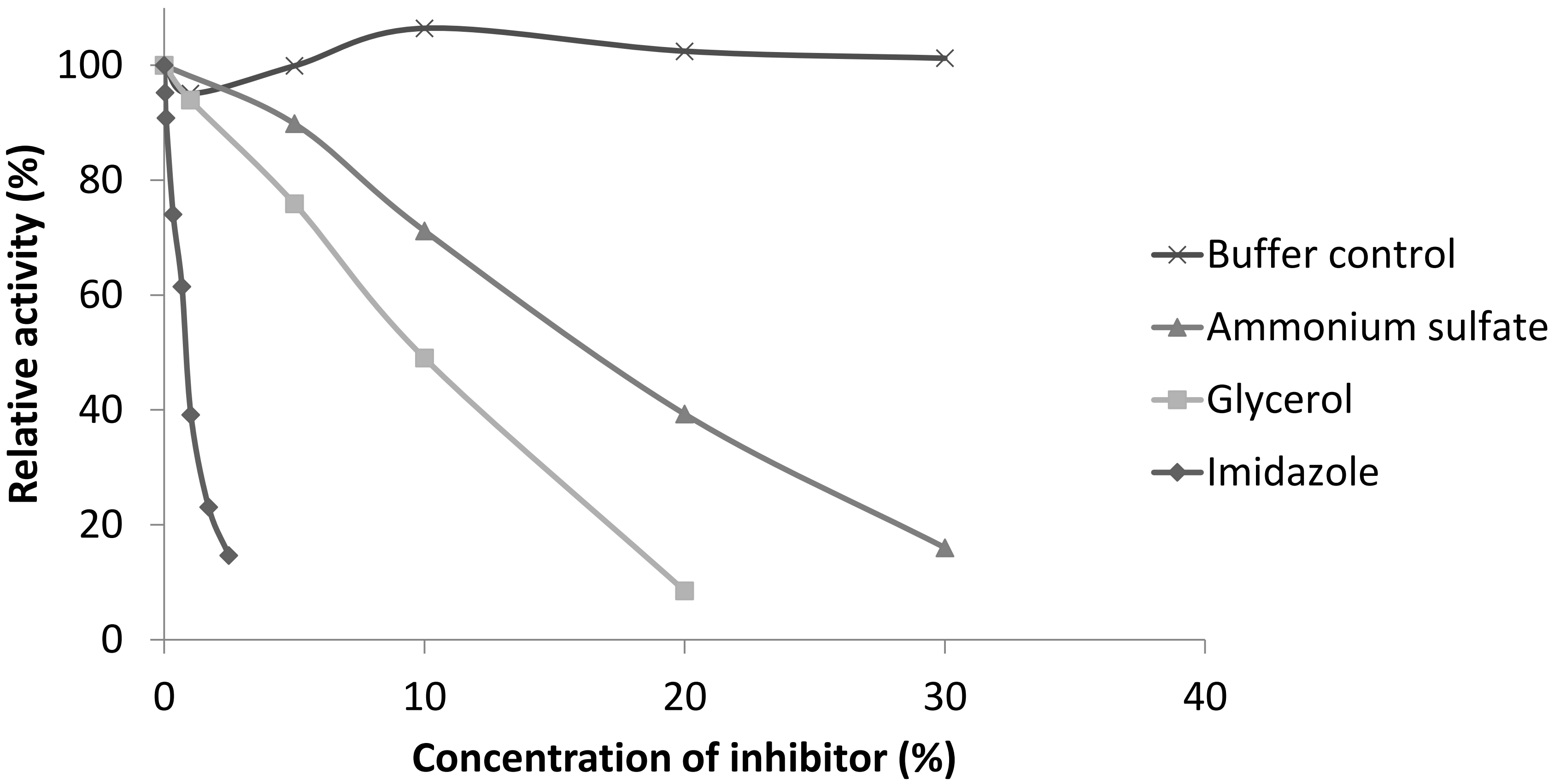

Supplement: Supplementary file 3 — Additional file 3. Effect of inhibitors on the cellulosomalcomplexes. The glucose concentration was measured using the Glu-HK determination kit (Megazyme). The residual complex activity was assessed using 0.5 mL standard reaction mixture containing 0.25% (w/v) of the substrate for one to 2 days of incubation at 60 °C. The initial activity at time point 0 corresponds to 100% relative activity. [file 13068_2018_1220_MOESM3_ESM.pdf]
