# Supplementary material for: Optimizing the composition of a synthetic cellulosome complex for the hydrolysis of softwood pulp: identification of the enzymatic core functions and biochemical complex characterization
Source: Biotechnol Biofuels. 2018 Aug 9;11:220. doi: 10.1186/s13068-018-1220-y (PMC6083626; doi:10.1186/s13068-018-1220-y)
Supplement: Supplementary file 4 — Additional file 4. Overview of 47 recombinant proteins. All proteins were mixed in equimolar amounts before adding them to the pentavalent SKLMY complex. [file 13068_2018_1220_MOESM4_ESM.docx]

**Additional file S4: Overview of 47 recombinant proteins.** All proteins were mixed in equimolar amounts before adding them to the pentavalent SKLMY complex.

|  |  |  |
| --- | --- | --- |
| **Locus tag** | **molecular weight (kDa)** | **molar ratio on CipA8** |
|  |  |  |
| Cthe_0071 | 94 | 0.16 |
| RS11245 | 79 | 0.16 |
| RS11155 | 67 | 0.16 |
| RS11085 | 59 | 0.16 |
| RS10750 | 12 | 0.16 |
| RS10235 | 36 | 0.16 |
| RS09970 | 49 | 0.16 |
| RS09915 | 52 | 0.16 |
| RS09910 | 54 | 0.16 |
| RS09185 | 59 | 0.16 |
| RS09150 | 100 | 0.16 |
| RS09145 | 138 | 0.16 |
| RS09035 | 40 | 0.16 |
| RS08595 | 64 | 0.16 |
| RS08560 | 82 | 0.16 |
| RS08380 | 82 | 0.16 |
| RS08095 | 178 | 0.16 |
| RS08090 | 82 | 0.16 |
| RS08010 | 65 | 0.16 |
| RS07895 | 87 | 0.16 |
| RS07890 | 64 | 0.16 |
| RS07470 | 82 | 0.16 |
| RS07205* | 54 | 0.16 |
| RS07200 | 58 | 0.16 |
| RS07080 | 61 | 0.16 |
| RS06630 | 117 | 0.16 |
| RS05040 | 75 | 0.16 |
| RS04370 | 90 | 0.16 |
| RS04360 | 47 | 0.16 |
| RS11350 | 102 | 0.16 |
| RS12825 | 67 | 0.16 |
| RS13380 | 92 | 0.16 |
| RS13665 | 90 | 0.16 |
| Clo1313_2747 | 83 | 0.16 |
| RS14195 | 65 | 0.16 |
| RS14250 | 74 | 0.16 |
|  |  |  |
| **Locus tag** | **molecular weight (kDa)** | **molar ratio on CipA8** |
|  |  |  |
| RS14510 | 100 | 0.16 |
| RS14525 | 106 | 0.16 |
| RS14530 | 57 | 0.16 |
| RS00915 | 72 | 0.16 |
| RS02020 | 67 | 0.16 |
| RS02025 | 67 | 0.16 |
| RS02085 | 63 | 0.16 |
| RS02120 | 55 | 0.16 |
| RS02540 | 59 | 0.16 |
| RS02665 | 72 | 0.16 |
| RS02880 | 68 | 0.16 |
|  |  |  |
|  |  |  |
|  |  |  |
